# Nurseped: educational technology for safety in the management of intravenous antibiotics in pediatrics *

**DOI:** 10.1590/1518-8345.6886.4068

**Published:** 2023-12-04

**Authors:** Amanda Paiva Bernardes Alves, Natália Del’ Angelo Aredes, George Oliveira Silva, Faétila dos Santos Oliveira, Luciana Mara Monti Fonseca, Laiane Medeiros Ribeiro

**Affiliations:** 1 Universidade Federal de Goiás, Goiânia, GO, Brasil; 2 Becario de la Coordenação de Aperfeiçoamento de Pessoal de Nível Superior (CAPES), Brasil.; 3 Universidade Federal de Goiás, Faculdade de Enfermagem, Goiânia, GO, Brasil.; 4 Becaria del Conselho Nacional de Desenvolvimento Científico e Tecnológico (CNPq), Brasil.; 5 Universidade de São Paulo, Escola de Enfermagem de Ribeirão Preto, Centro Colaborador de la OPS/OMS para el Desarrollo de la Investigación en Enfermería, Ribeirão Preto, SP, Brasil.; 6 Universidade de Brasília, Faculdade de Ciências da Saúde, Brasília, DF, Brasil.

**Keywords:** Patient Safety, Pediatric Nursing, Medication Errors, Educational Technology, Nursing Students, Nursing, Seguridad del Paciente, Enfermería Pediátrica, Errores de Medicación, Tecnología Educacional, Estudiantes de Enfermería, Enfermería, Segurança do Paciente, Enfermagem Pediátrica, Erros de Medicação, Tecnologia Educacional, Estudantes de Enfermagem, Enfermagem

## Abstract

**Objective:**

to develop and validate the content of a serious game on the safe management of intravenous medications in pediatrics.

**Method:**

methodological study for the development and content validation of an educational technology. The cases and challenges of the serious game were developed based on a literature review and validated by 11 nurses with training and experience in the area. Content validity and agreement indices were adopted to analyze agreement and internal consistency (minimum of 0.8).

**Results:**

the content is based on the main antibiotics used in the clinical management of infections in hospitalized children and patient safety. Absolute agreement was obtained in 60 of the 61 items evaluated, and the minimum obtained was 0.82 in the content validation index and 0.80 in agreement. Adjustments were suggested by experts in the response statement for a specific case and implemented to improve the quality of the technology content.

**Conclusion:**

the content of the serious game Nurseped was validated by nurse experts in child health regarding clinical cases, question statements and multiple-choice answers, in addition to feedback that presents the user with an evidence-based answer after getting the challenge right or wrong.

## Abstract (es)

**Objetivo:**

desarrollar y validar el contenido de un *serious game* sobre el manejo seguro de medicamentos intravenosos en pediatría.

**Método:**

estudio metodológico para el desarrollo y validación de contenido de una tecnología educativa. Los casos y desafíos del *serious game* se basaron en una revisión de la literatura y fueron validados por 11 enfermeros con formación y experiencia en el área. Se adoptaron índices de validez de contenido y concordancia para el análisis de concordancia y consistencia interna (mínimo de 0,8).

**Resultados:**

el contenido se basa en los principales antibióticos utilizados en el manejo clínico de infecciones en niños hospitalizados y en la seguridad del paciente. Se obtuvo una concordancia absoluta en 60 de los 61 ítems evaluados, y el mínimo obtenido fue de 0,82 en el índice de validación de contenido y 0,80 en concordancia. Los expertos sugirieron ajustes en la formulación de respuestas de un caso específico y se implementaron para mejorar la calidad del contenido de la tecnología.

**Conclusión:**

el contenido del *serious game Nurseped* fue validado por enfermeros expertos en salud infantil en cuanto a casos clínicos, enunciados de preguntas y respuestas de opción múltiple, además del *feedback* que presenta al usuario una respuesta basada en evidencia tras acertar o fallar en el desafío.

## Abstract (pt)

**Objetivo:**

desenvolver e validar o conteúdo de um *serious game* sobre o manejo seguro de medicamentos endovenosos em pediatria.

**Método:**

estudo metodológico para o desenvolvimento e validação de conteúdo de uma tecnologia educacional. Os casos e desafios do *serious game* foram desenvolvidos com base em revisão da literatura e validados por 11 enfermeiros com formação e atuação na área. Adotou-se os índices de validade de conteúdo e concordância, para análise de concordância e consistência interna (mínimo de 0,8).

**Resultados:**

o conteúdo é baseado nos principais antibióticos utilizados no manejo clínico de infecções em crianças hospitalizadas e na segurança do paciente. Obteve-se concordância absoluta em 60 dos 61 itens avaliados, o mínimo obtido foi 0,82 no índice de validação de conteúdo e 0,80 na concordância. Ajustes foram sugeridos pelos *experts* no enunciado de respostas de um caso específico e implementados para o aprimoramento da qualidade do conteúdo da tecnologia.

**Conclusão:**

o conteúdo do *serious game* *Nurseped* foi validado por enfermeiros *experts* em saúde da criança quanto aos casos clínicos, aos enunciados das perguntas e às respostas de múltipla escolha, além do *feedback* que apresenta ao usuário uma resposta baseada em evidências após o acerto ou erro do desafio.

**Highlights:**

**(1)** The content of the serious game Nurseped was validated by expert pediatric nurses.

**(2)** The serious game can contribute to training and continuing education in nursing.

**(3)** Technology can increase the quality of care for hospitalized children.

## Introduction

In addition to the use of digital technologies to support direct health care and support the interoperability of existing systems in the Unified Health System (SUS), strengthening the education of human resources in digital health is part of the list of priorities of the Health Digital Strategy for Brazil ^(^ 1 ^)^ . Learning mediated by digital technologies is part of this context by enabling experiences of human-computer interaction, bringing students and health professionals closer to the technology itself while enabling the development of new knowledge and skills in health from a computational tool ^(^ 2 ^-^ 3 ^)^ . 

Faced with a gap identified in the training process of future nurses and nursing technicians, and in clinical practice in the area of child health, especially in the hospital environment ^(^ 4 ^)^ , an educational technology of the serious game type was developed to support the process of teaching-learning of students and nursing professionals in the safe management of medications, with emphasis on the preparation and administration of intravenous antibiotics, which represent relevant indicators in the emergence of venous access complications ^(^ 5 ^)^ . 

Such management involves the processes of preparing and administering medication, a daily activity performed by nursing teams in pediatric units and responsible for considerable rates of adverse events ^(^ 6 ^-^ 7 ^)^ . A retrospective study in a hospital unit in the United States of America showed, after evaluating 3,790 records, a rate of 9.5 preventable adverse events per 1,000 patients/day ^(^ 6 ^)^ . In Brazil, the scenario of notifications highlights drug administration as the main cause of adverse events in pediatrics, representing 65.6% of all notifications between 2007 and 2013 ^(^ 7 ^)^ . 

It is estimated that the occurrence of medication errors in children is more frequent compared to adults, reaching three times higher, resulting in variations in the type of error ranging from the prescribed dosage to dilution, and an increased risk for the patient, as children have different physiological characteristics, such as body immaturity, a wide weight range, and different development stages than other populations, such as adults and the older adults ^(^ 8 ^)^ . 

To transform this adverse scenario, in search of strengthening patient safety in pediatrics, it is necessary to provide nursing professionals with mechanisms and tools aimed at learning from the initial stage of their professional training ^(^ 9 ^)^ , especially related to the adoption of barriers to safety in the preparation and administration of medicines in pediatrics, such as cleaning the preparation site, disinfecting the ampoule, connecting, checking the medicine/dose/route of administration with the prescription and double checking the medicines ^(^ 10 ^)^ . Still, such tools need to consider the different stages of drug management, based on patient safety, and address the calculation skills for dosage confirmation, reconstitution (in which nursing extracts pediatric doses from bottles produced to handle adult doses), dilution and dripping or flow ^(^ 11 ^)^ . 

Adding the challenge of recording adverse events related to drug administration in clinical practice in pediatrics, the need to improve initial and continuing nursing education on the subject, and strategies to leverage digital health ^(^ 1 ^)^ , the serious game Nurseped proposes to support the teaching-learning process through challenges and evidence from updated scientific literature, combining entertainment and knowledge in a digital educational technology. Thus, this study aims to develop and validate the content of a serious game on the safe management of intravenous medications in pediatrics. 

## Method

### Type of study

Methodological study ^(^ 12 ^)^ was developed containing the initial stages of development of a serious game, namely, 1) Literature review, 2) Preparation of clinical challenges, and 3) Seriou’s game content validation Nurseped. The present study was reported according to the SQUIRE 2.0 criteria (Standards for Quality Improvement reporting Excellence), checklist for reporting studies focused on improving health care ^(^ 13 ^)^ . 

### Location, sample and inclusion criteria

The validation process was carried out entirely in an online environment, with experts from different locations in the country, provided they met the study inclusion criteria: being a graduated nurse with clinical experience in child health, obtaining at least five points according to the specific reference for validation studies ^(^ 14 ^)^ ( Figure 1). 

**Figure 1 -:** Criteria and score for defining experts for content validation in health technologies. Goiânia, GO, Brazil, 2022-2023

Criterion	Points
Clinical experience of at least 4 years in the field of child health	4 points
PhD title	2 points
Master’s degree	1 point
Publication in a journal in the field of nursing	1 point
Participation of at least 2 years in research or research group in child health	1 point
Nursing residency in the area of child health	1 point
At least 1 year of experience in teaching child health	1 point

The study sampling process was for convenience, with the selection of potential participants with recognition in the area of child health (care or teaching/research), who were identified through searches on the Lattes platform via the National Council for Scientific and Technological Development – CNPQ or indicated by the experts who initially agreed to participate in the study, through the “snowball” method, which expanded the recruitment potential. Thus, 58 nurses with the potential to participate in the study were identified, so that all were invited via e-mail. The form with the data collection instrument was available online between November 2022 and March 2023.

After the established deadline, 12 respondents were identified, representing a response rate of 20.7%, who were screened according to the inclusion criteria. One participant was excluded for not reaching the minimum required score (5 points), although he had completed and signed the ICF, and contributed to the analysis of the challenges. Thus, the final study sample consisted of 11 experts.

### Serious game development

Heuristic was adopted as a methodological reference for developing the serious game. Evaluation for Digital Educational Games (HEDEG) ^(^ 15 ^)^ , a reference that establishes a set of heuristics for the development of educational games, namely: i) interface (IN): elements that establish communication between the student and the game environment; ii) educational elements (ED): which evokes the acquisition of knowledge by students; iii) content (CN): which refers to the disciplinary contents on which the game is based and for which the student’s development is desired; iv ) gameplay (JG): consisting of experience and interaction with the game; v) multimedia (MM): consisting of elements that make up the game’s media, such as sounds, images and videos. 

This study presents the content validation step of the serious game Nurseped, so the development, testing and implementation steps will be presented in subsequent studies. In order to visualize how the validated cases will be presented in the serious game, a prototype was created in the software Canva, containing images and colors according to the children’s theme, demonstrating the sequence of screens and representing the navigation of the developed and validated content.

### Data collection

For the validation of the content of the serious game, cases and challenges related to them were prepared, based on a literature review carried out in two stages: 1) analysis of protocols for the preparation and administration of antibiotics in pediatrics on the official websites of reference institutions for care pediatric hospital, and 2) analysis of medication package inserts and scientific articles on the subject. Free searches were performed in the Medline databases (Medical Literature Analysis and Retrieval System Online) via PubMed, LILACS (Latin American and Caribbean Health Sciences Literature) and Google Scholar, using non-standard terms related to antibiotics. The limit of antimicrobials was defined from the universe of drugs used in the treatment of hospitalized children, considering the frequency of use of the class of drugs and the risk of adverse events in the studied population.

The search for scientific literature and the protocols of reference institutions for in-hospital pediatric care supported the elaboration of clinical cases with consistent adequacy of medications to the children’s ages, preparation recommendations and guidelines for safe administration. This data collection format on the subject made it possible to add important information from the moment of prescription related to calculations and surveillance of interactions, to monitoring after administration, considering clinical evaluation and good safety practices.

From the identification of the main antibiotics used in pediatrics, plausible clinical challenges were prepared with situations of clinical practice in child care. The challenges were later sent for analysis by the experts, available in the following format: a) Brief clinical case containing the prescription of the antibiotic; b) Drug data (minimum and maximum daily dose, form of drug presentation, diluent, final concentration for infusion and infusion time by age group); c) questions and correct answers for each mathematical challenge and d) clinical challenge containing a situation likely to occur with an option for evidence-based feedback (examples: flushing technique, visual interpretation of syringe graduations, care with peripherally inserted central catheters and periodicity or criteria for changing venous access in children).

In each validation module, on Google Forms, a Likert scale was presented to assess the clinical challenges in view of the variables: clinical relevance, relevance of learning on the topic, alignment with the scientific literature, and clarity of the statement. The Likert scale and the scores that guided the statistical analysis ranged from 1 to 4 points, as follows: I disagree (1), I suggest major changes (2), I suggest small changes (3) and I agree (4).

### Data analysis

The data were tabulated in the software Statistical Package for the Social Sciences (SPSS) version 29. For the analysis, the Content Validity Index (CVI) was used in order to assess the concordance index between the experts’ answers *,* and the items were considered validated when greater than 0.80. The CVI evaluates the representation of each item on a four-point Likert-type scale, ranging from disagree (“1”) to agree (“4”). The score was obtained by adding the agreement of the items marked with a score of “3” or “4” by the specialists and divided by the total number of responses, multiplied by 100 (CVI = agreement with a score of “3” or “4”/total responses x 100). Items with scores of “1” or “2” were mandatory revised. 

Agreement between experts was verified using the Modified Kappa Coefficient (MKC) ^(^ 16 ^)^ . To calculate the MKC, the probability of chance of agreement for each item was calculated using the formula:

\begin{equation*}P_C = [N! /A! (N -A)!]^* .\ 5^N\end{equation*}

Where, N = number of *experts*, A = number of *experts* who agree that that item is relevant. The calculation of the MKC was performed from the probability of chance of agreement and the CVI of each item, using the formula:

\begin{equation*}MKC = (IVC - P_C)\ /\ (1- P_C)\end{equation*}

Where, P _C_ = numerical chance probability agreement values. As a reference for the cut-off point, we adopted: MKC between 0.40 and 0.59 as weak agreement; MKC between 0.60 and 0.74 as good agreement; and MKC above 0.75 as excellent agreement ^(^ 16 ^)^ . 

### Ethical aspects

For the validation stage, approval was obtained from the Research Ethics Committee, opinion No. 5,208,367, so that all stages of the study complied with the recommendations for research with human beings. The Informed Consent Form (ICF) was attached to the invitation letter sent to the experts by e-mail and, once they voluntarily consented to participate and so indicated in the document, they were directed to a page with the data collection instrument.

## Results

Among the many possibilities of drugs, the sample was defined as the main antibiotics administered intravenously in children, reaching a total of 19 drugs identified in the literature review, the main ones being: Amikacin; Ampicillin; Cephalotin; Cefazolin; Cefepime; Ceflozadime; Cefotaxime; Ceftriaxone; Cefuroxime; Clarithromycin; Clindamycin; Chloramphenicol; Gentamicin; Meropenem; Oxacillin; Pyreracillin+Tazobactan; Polymyxin B; Sulfatoxazole+Trimetropin; Teicoplanin and Vancomycin.

From the identified drugs, information was extracted about name and pharmacological class, indications for use, routes of administration, presentation and recommended dose for children, infusion time, preparation guidelines, incompatibility and drug interactions, and adverse events. This information supported the elaboration of the content so that the serious game reflects situations to which nurses are exposed on a daily basis when working with medication management in pediatrics.

Thus, 12 clinical challenges were created, containing activities to calculate dosage, dilution, and dripping, and activities to strengthen good practices in the process of administering medication to children, such as the game of 7 errors and questions related to the interaction of fluids, biosafety procedures, and nursing. The prepared content was subsequently submitted to validation by experts, considering that this process is recommended for studies aimed at developing of educational technologies ^(^ 17 ^)^ , in order to guarantee quality and reach higher levels of scientific reliability, essential attributes for digital health. 

Eleven experts from the South, Southeast, and Center -West regions of Brazil participated in the content validation proposed in this study. Regarding characterization, the *experts* were nurses (and one nurse) with a minimum age of 28 and a maximum of 50 (mean = 34.08 years; standard deviation (SD) = 7.55), working mainly in child health care (n=5; 45.45%), followed by teaching and research (n=2; 18.18%). One *expert* associated assistance and teaching (9.09%), just as this frequency appears the same for those who are working exclusively in research and combining research and management at the moment. 

The scores obtained by the experts, according to Guimarães, et al. ^(^ 14 ^)^ , ranged from 5 to 10, with a mean equal to 7.45 and SD of 1.50 points. It should be noted that clinical experience is responsible for most of the points, valuing the knowledge of nurses in the practice of care, in addition to training as a specialist, master and doctor, as shown in Table 1. 

**Table 1 -:** Titles presented by specialist nurses selected in the study. Goiânia, GO, Brazil, 2022-2023

Criteria	Frequency	Percentage
Clinical experience of at least 4 years in the field of child health	10	90.9%
PhD title	3	27.7%
Master’s degree	7	72.72%
Publication in a journal in the field of nursing	9	81.81%
Participation of at least 2 years in research or research group in child health	7	63.63%
Nursing residency in the area of child health	5	54.54%
At least 1 year of experience in teaching child health	7	54.54%

Although only one expert mentioned previous experience with the development of educational games, the expressive majority *,* in addition to assisting in child health, have a history of publications in nursing journals, and a training profile in both *lato sensu* and *stricto sensu* postgraduate courses, denoting association of practice with continued education and engagement in academia. Just over half (n=6; 54.54%) had already played an educational game at the time of data collection. 

The CVI, analyzed through the experts’ responses, received a value greater than 0.80 in all topics submitted to evaluation. In this way, the content of the serious game Nurseped was considered validated with an excellent rating. The themes of the cases and the results of content validation and concordance are shown in Table 2. 

**Table 2 -:** Aspects evaluated in the validation instrument of clinical cases by specialists and analysis of CVI and CKM. Goiânia, GO, Brazil, 2022-2023

Clinical case linked to the item	Rated item	IVC ^*^	CKM ^†^	Classification
Case 1 – Installation of antibiotics and other fluids in CIP ^‡^ (Amicacin)	Clinical case	1.00	1.00	Great
	Prescription and presentation of drug data	1.00	1.00	Great
	Statements of questions	1.00	1.00	Great
	Answers	1.00	1.00	Great
	Feedback	1.00	1.00	Great
Case 2 – Installation of antibiotics and other fluids in CIP ^‡^ (Vancomycin)	Clinical case	1.00	1.00	Great
	Prescription and presentation of drug data	1.00	1.00	Great
	Statements of questions	1.00	1.00	Great
	Answers	1.00	1.00	Great
	Feedback	1.00	1.00	Great
Case 3 – Installation of antibiotics and other fluids in CIP ^‡^ (Sulfamethoxazole)	Clinical case	1.00	1.00	Great
	Prescription and presentation of drug data	1.00	1.00	Great
	Statements of questions	1.00	1.00	Great
	Answers	1.00	1.00	Great
	Feedback	1.00	1.00	Great
Case 4 – Installation of antibiotics and other fluids in CIP ^‡^ (Ceftriaxone)	Clinical case	1.00	1.00	Great
	Prescription and presentation of drug data	1.00	1.00	Great
	Statements of questions	1.00	1.00	Great
	Answers	1.00	1.00	Great
	Feedback	1.00	1.00	Great
Case 5 – Installation of antibiotics and other fluids in CIP ^‡^ (Oxacillin)	Clinical case	1.00	1.00	Great
	Prescription and presentation of drug data	1.00	1.00	Great
	Statements of questions	1.00	1.00	Great
	Answers	0.82	0.80	Great
	Feedback	1.00	1.00	Great
Case 6 – Amphotericin B and administration of antibiotics and other fluids in PICC ^§^	Has clinical relevance	1.00	1.00	Great
	It has learning relevance in the theme	1.00	1.00	Great
	Aligns with scientific literature	1.00	1.00	Great
	statement is clear	1.00	1.00	Great
	Question format is adequate	1.00	1.00	Great
Case 7 – Antibiotic therapy and total parenteral nutrition	Has clinical relevance	1.00	1.00	Great
	It has learning relevance in the theme	1.00	1.00	Great
	Aligns with scientific literature	1.00	1.00	Great
	statement is clear	1.00	1.00	Great
	Question format is adequate	1.00	1.00	Great
Case 8 – Administration of blood components in PICC ^§^	Has clinical relevance	1.00	1.00	Great
	It has learning relevance in the theme	1.00	1.00	Great
	Aligns with scientific literature	1.00	1.00	Great
	statement is clear	1.00	1.00	Great
	Question format is adequate	1.00	1.00	Great
Case 9 – Evaluation of phlogistic signs in peripheral venous access	Has clinical relevance	1.00	1.00	Great
	It has learning relevance in the theme	1.00	1.00	Great
	Aligns with scientific literature	1.00	1.00	Great
	statement is clear	1.00	1.00	Great
	Question format is adequate	1.00	1.00	Great
Flushing procedure	Has clinical relevance	1.00	1.00	Great
	It has learning relevance in the theme	1.00	1.00	Great
	Aligns with scientific literature	1.00	1.00	Great
	statement is clear	1.00	1.00	Great
	Question format is adequate	1.00	1.00	Great
Case 11 – Exchange of venous access	Has clinical relevance	1.00	1.00	Great
	It has learning relevance in the theme	1.00	1.00	Great
	Aligns with scientific literature	1.00	1.00	Great
	statement is clear	1.00	1.00	Great
	Question format is adequate	1.00	1.00	Great
Case 12 – Prevention of bloodstream infection	Dosage calculation for children	1.00	1.00	Great
	Drip and flow calculation	1.00	1.00	Great
	Safe medication preparation	1.00	1.00	Great
	Safe medication administration	1.00	1.00	Great
	evidence-based	1.00	1.00	Great
	Aligned with quality nursing care for hospitalized children	1.00	1.00	Great

^*^CVI = Content Validity Index; ^†^MKC = Modified Kappa Coefficient; ^‡^CIP = Continuous Infusion Pump; ^§^PICC = Peripherally Inserted Central Catheter

More than obtaining at least 0.8 in the CVI in all items, it should be noted that only one item was not assigned the maximum score (1.0), which corresponds to clinical case 5 in the item “responses”. In this, six experts (54.54%) suggested minor changes, and two (18.18%) suggested major changes, which refer to the way of writing.

The suggestions were incorporated and occurred for the standardization of acronyms, and correction regarding the dose of the right answer. One of the suggestions was very important for the safety of the hospitalized child, regarding the venous access flushing procedure: “As for the feedback on the extra question, remember that it is not necessary to use 10 ml of 0.9% saline solution to wash the access, especially if there is water restriction” (Expert 2).

There were also changes in the clinical case regarding the concentration required for dilution, as suggested by the experts, as a specific volume is required for reconstitution, considering the milligrams of the presentation dose, the volume and the final concentration for intravenous infusion. The correct thing, suggested and followed, is to consider the aspirated volume during the dilution, to be incorporated into the reconstitution solution, that is, if the maximum expected concentration for the 100 mg dose of a given antibiotic prescribed for the patient is 2 mg/ml, the volume for reconstitution must consider the aspirated volume of antibiotic content as the final volume.

Other adjustments were suggested and enabled textual improvement, contemplating the way the statements were written, the explanatory feedback, and, in particular, allowed advancing in the addition of important content to the learning process about the safe handling of medication in children. One example was to incorporate the consequences of errors in drug administration into the clinical case feedback, the importance of knowing drug interactions, and reinforce the relevance of dose and concentration calculations, all from the perspective of patient safety, under the recommendations of the “medication rights”, currently described in 9 rights ^(^ 18 ^)^ . 

**Figure 2 -:**
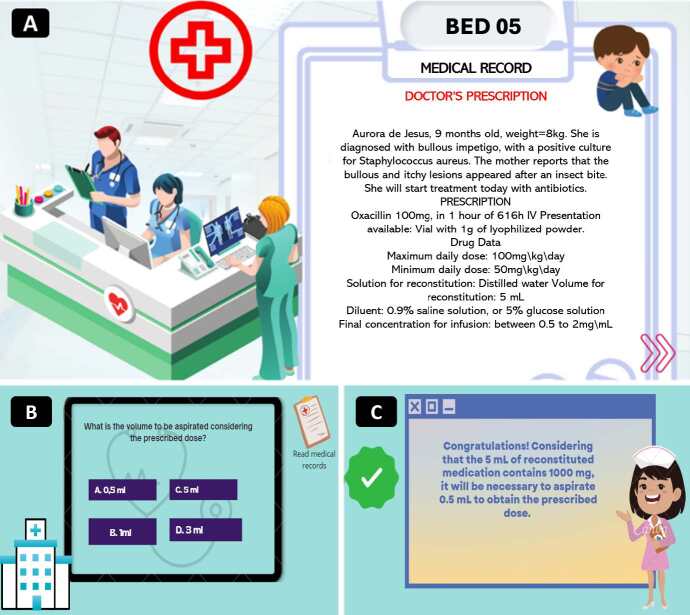
Clinical case 5 of the serious game Nurseped. Goiânia, GO, Brazil, 2022-2023

After the suggested adjustments, a prototype of the serious game interface was created with case 5 presentation screens, the question with answer options and the feedback provided to the player ( Figure 2). The first phase of the serious game consists of the game of errors and continues with the calculation challenges and other safety aspects in the management of intravenous drugs in pediatrics. The player chooses a clinical case (among the 12 developed and validated), which will offer a medical prescription to be executed and extra challenges per case: dose calculation, drip infusion calculation and/or flow rate in a continuous infusion pump and questions about administration of medicines in special situations of care for hospitalized children. 

A) Description of the case; B ) Question and answer options; C) Response feedback

## Discussion

The serious game Nurseped was considered validated by the experts regarding its content and is still in the production phase, since it adheres to scientific evidence, has textual clarity and relevance for the teaching-learning process, aligning itself with quality nursing care for children hospitalized in the topic of safe medication management.

Serious game format was chosen for combining the possibility of developing knowledge and providing entertainment, containing the basic elements of an educational game in a computational environment: levels, points, avatars, tips, multimedia and challenges to be solved in a certain time ^(^ 19 ^)^ . That is, in addition to the gamification that uses the engagement structure of a game, but does not have all the necessary elements ^(^ 20 ^)^ , Nurseped includes all the elements that define it as a serious game and can contribute to the education of students and staff nursing staff who provide hospital care to children. 

Some studies highlight the advantages of using serious games as an educational support tool in nursing, including the potential for engagement and motivation ^(^ 21 ^)^ , satisfaction ^(^ 22 ^)^ and improved performance based on learning ^(^ 23 ^-^ 24 ^)^ . In this way, the development of these technologies for use in professional training courses in nursing and in continuing or permanent health education, depending on the applied context, is an important step in digital health ^(^ 2 ^)^ . 

Another context in which serious games are applied is through health education. Recent studies, in the area of child health from the perspective of digital health, point to the potential of serious games to promote changes in behavior and development of relevant knowledge for children and their families or guardians ^(^ 25 ^-^ 26 ^)^ . 

Regarding the format of serious games, a recent systematic review of the nursing literature identified several possibilities, not restricted to 3D (three-dimensional) virtual simulations of greater technological complexity ^(^ 17 ^)^ . It was concluded that although the use of high-end technology can produce better entertainment effects, simpler serious games with quizzes as is the case of Nurseped, and modifications of existing systems for managing the teaching-learning process, can be equally effective for nursing education. 

Game-based learning is a method considered effective to improve nursing students’ cognitive learning on different topics ^(^ 19 ^)^ . The same authors who reach this conclusion after systematic review and analysis of 47 studies call attention to the plurality of countries that have already reported this type of initiative in scientific studies (in North America, Europe, Oceania and Asia), including Brazil, as only representative of Latin America in the research ^(^ 24 ^)^. 

It should be noted that despite the multiplicity of game format options, platforms, gamification elements, themes and objectives, one aspect is fundamental in all of them to guarantee quality, scientific basis and relevance for the teaching-learning process: validation with experts regarding the content. Other validations are important, such as interface ^(^ 27 ^-^ 28 ^)^ and usability ^(^ 28 ^-^ 29 ^)^ , but this study reinforces the importance of content validation. 

This highlight is due to the importance of moving forward with digital health in Brazil ^(^ 1 ^)^ , and the need to guarantee health data security, quality of information conveyed in times when access is wide, but especially the guarantee of veracity, quality and scientific basis do not represent the total of materials and publications available for public access ^(^ 30 ^-^ 31 ^)^ . 

Furthermore, in the context of technologies for educational purposes, the theoretical-pedagogical framework is a very relevant topic. The reference adopted by teachers when devising and using a *serious game* or other tool for educational purposes can be revealed: 1) in the navigation dynamics and in the content of the feedbacks themselves (tool development), and 2) in the application of this tool to the process of teaching-learning, which is, in a broader perspective, the sum of the teaching plan and tool with the teacher’s pedagogical approach related to the way of using the tool and application of the teaching plan. 

Although there are limitations to reflect the complexity of theoretical frameworks in the dynamics of a serious game, these can manifest themselves in different situations. For example, the approach in which the user can choose their avatar freely and the error or success *feedback* is welcoming and encourages them to continue in the game, without blocking the evolution in the game due to the eventual accumulation of errors, for example, is aligned with humanism. and without punishing with the need to go back to the beginning ^(^ 15 ^)^ . Another situation that can be exemplified is the use of challenges based on cases that appear in clinical practice and that represent a real problem, approaching problematization. 

An interesting model for the area of development of educational technologies for nursing, based on reflection on the theoretical basis in the field of pedagogy, is the cognitive approach model in the elaboration of tools that aim to encourage the learning of clinical reasoning ^(^ 32 ^)^ . The presented model brings the elements used in face-to-face education that permeate the act of teaching, the addition of difficulty levels, the teaching-learning activities and the teacher’s example of what would be ideal, linking them to characteristics of educational technologies digitalis, as detailed below ^(^ 32 ^)^ . 

They are: interactive technology support; questioning and forum/debriefing (applicable for technologies with teacher mediation, that is, non-self-instructional); automated feedback; trial and error procedures; formative assessment throughout the interaction with the tool; educational activities and gamification; and presentation of solved cases, examples and visualization of the clinical reasoning process ^(^ 32 ^)^ . 

In the case of the serious game validated in this study, in addition to the logical reasoning evoked for the calculation and dosage of medications, clinical reasoning is guided by the nurses’ cognitive steps to arrive at the safest and most appropriate intervention for each case of medication management. Clinical reasoning, being a complex cognitive process that uses formal and informal thinking strategies and analyzes available data on clinical cases to propose the best intervention ^(^ 32 ^)^ , includes different skills regarding the safe management of medication in pediatrics. 

It is extremely important to encourage innovative teaching methods, attitudes that lead the student to a critical reflection on the practice, as they must acquire knowledge of the cognitive and technical aspects as future nurses ^(^ 33 ^-^ 35 ^)^ . It is necessary to guarantee education to nurses, since the undergraduation, demonstrating that they are fundamental in the management of services, and are directly linked to the management of health care. Therefore, it is evident that education that promotes greater autonomy, self-reflection and self-criticism guarantees future improvement in the nursing team, leading to greater chances of quality care, a safe environment and professional qualification ^(^ 36 ^)^ . 

Considering that this study presents the initial stage of developing the serious game, it was limited only to presenting the content validation stage with experts, so the other stages will be presented in subsequent studies. Other limitations of the study were the low adherence of nurses to compose the panel of experts, considering the response rate when sending the invitations, and the non-participation of undergraduate students in this validation stage to assess the understanding of the terms used in the cases, phase that will be implemented in the test phase of the serious game.

Based on the content created for the serious game and the indicators available in the literature, this study intends to contribute to the education of nurses both at the undergraduate and continuing education levels, considering that the prevention and reduction of care errors is a fundamental part of care of nursing. This purpose is even more relevant considering that the target population of care is more vulnerable, such as neonates and children, in which dosage errors and gaps in patient safety knowledge can be fatal or cause serious harm.

Thus, the present study contributes to the advancement of scientific knowledge, in the context of information and communication technologies, to the strengthening of nursing education, with the aim of impacting reality, increasing the quality of care and good safety practices for the patient. patient for hospitalized children and their families. In addition, it is expected that the technology developed when applied to active teaching-learning strategies in different contexts of nursing education can contribute to the achievement of patient safety goals, with emphasis on the safe administration of medications.

## Conclusion

The serious game Nurseped, developed to support the teaching-learning process of nursing students and nursing staff working in pediatrics on the issue of safe management of intravenous antibiotics in hospitalized children, was validated by experts. Validation was obtained regarding clinical and learning relevance, alignment with scientific literature, clarity and format of questions, response options and feedback offered to the user. All items obtained a CVI and MKC of at least 0.8, classified as excellent in terms of consistency and agreement in the evaluation of the 11 participants, with only one case not obtaining the maximum score, although it obtained the established minimum.

In addition, in the qualitative aspect of the experts’ participation, the evaluations added a lot to the quality of the final version, with particularities of the pediatric area, so that the content is even closer to the clinical reality, which was enhanced by the profile of the experts and by the methodological framework adopted for selecting participants. In Brazil, there are still few digital educational tools developed and validated to strengthen nursing education, especially in the pediatric area. This gap is even more evident in scientific studies and demonstrates that this is a scenario yet to be explored in the country.
